# Lectures on Anatomy: Interspersed with Practical Remarks. Vol. I

**Published:** 1830-01-01

**Authors:** 


**Lectures on Anatomy: interspersed with Practical Remarks. Vol. I. By *B. B. Cooper*, F.R.S. Surgeon of Guy's Hospital, Lecturer on Anatomy, &c. &c. &c. Royal Octavo, pp. 310. London, 1829.**

The number of works upon anatomy at present pouring from the press, argues an increasing taste or necessity for the study, despite of the impediments and difficulties that now beset it. We say an increasing call for anatomy, for no principle in political or other economy is better established than this, that the supply will always be regulated by the demand. There may indeed be an occasional glut, but a few bankruptcies or failures soon trim the balance. The great majority of the manuals and systems of anatomy or physiology of the present day are either avowedly or in reality taken from the French, and our students would seem to imagine, like our modern play-goers, that nothing whether large or small, opera or interlude, system or manual, can possibly be worth a fig unless it be imported from the other side of the water. God knows we share nothing in common with that Anti-Gallican, exclusive, and bigotted party whose gorges rise at the bare mention of French or Frenchman, and who tickle their John Bullish prejudices by holding all which is not their own in profound contempt. At the same time we are sufficiently patriotic to desire that in knowledge, as in war, we should not succumb before the sons of Gaul, but rather endeavour in generous rivalry to lead the van. It is on this account that we are disposed to hail the appearance of the present work on anatomy by Mr. Bransby Cooper with feelings of much pleasure, and we trust that the popular author of the Description of the Ligaments of the Human Body will add another and a greener leaf to the wreath of British writers that already adorn this part of our science.

The present volume merely embraces the anatomy of the bones and articulations, but it is our author's intention to publish another annually in each succeeding season, till the series shall thus be completed. Interspersed with the anatomical descriptions is a concise but accurate account of the physiology, morbid alterations, and injuries of the parts on which the lecturer is treating. It can scarcely fail to be perceived that although differing in toto from the plan of most of the foreign writers, the present must be attended with many advantages to the student. Of the execution we can speak in the most favourable terms, and we have little doubt that when completed, "Bransby Cooper's Anatomy" will become a class-book of much value and great circulation. Of course an analysis of an elementary book of this kind is perfectly out of the question, but the following extract will afford a sample, an imperfect one it is true, of the whole; it is an account of the fractures to which the os femoris is subject.

"*Practical Remarks.*

"Notwithstanding the great strength of this bone, still, as may be said of every cylindrical bone, it is liable to fracture in any part of its extent, either at the point of immediate application of a force, or it may give way in its centre, from a fall on the condyles; in which case the upper part of the bone forms the point d'appui, and the natural curvature of the bone has a tendency to be increased beyond its power of extension, and it necessarily yields. In consequence of the numerous muscles which are attached to this bone, it is obvious that the fractured extremities must vary in their direction, depending upon the influence of muscular power, and the precise point at which the femur is fractured; therefore we will consider individually those particular parts of the bone, which, when fractured, invariably take a certain direction.

" *Fracture of the Neck.—(Vide Fig.* 1, *Plate IX*).—It is unnecessary for me to offer any remarks upon the different opinions which surgical writers have entertained, concerning the union or non-union of this fracture. The diagnosis is always sufficiently distinct to point out the nature of the injury: first, this accident only occurs at an advanced period of life; the limb is shortened and everted; slight extension brings it to its natural length; and rotatory motion during extension produces crepitation. The only accident for which this can be mistaken, is dislocation upon the pubes; but the circumstance of the one being unnaturally fixed, while the other is capable of inordinate motion, will lead to a just knowledge of the injury. From the experience I have had upon this subject, I feel I cannot do better than recommend the practice adopted by Sir Astley Cooper, as laid down in his work on fractures and dislocations.

" *The Trochanter Major (Vide Fig.* 2, *Plate IX.)* is sometimes detached from the shaft of the bone:—an accident which it is difficult to detect, in consequence of the small size of the separated portion, and from its not producing any alteration in the direction or length of the limb; the fractured portion of bone being drawn up by the action of the m. gluteus medius and minimus, a considerable separation frequently occurs. In consequence of these two muscles being wholly inserted into the trochanter major, the adaptation of the fractured extremity is difficult both to produce and to maintain; and can only be accomplished by the abduction of the injured limb, in addition to the other ordinary means. I have had an opportunity of witnessing this accident, and experienced all the difficulties I have described.

" *Fractures immediately below the Trochanter Minor.—(Vide Fig.* 3, *Plate IX.)*—This accident differs from all other fractures of the thigh, excepting that last described, from the greater displacement occurring in the upper than the lower portion of the bone, in consequence of the insertion of the m. psoas magnus and iliacus internus the upper fractured extremity is drawn forwards, so as to form a tumour in the groin, which deformity can only be obviated by bending the pelvis upon the thigh, so that the patient must be placed in bed nearly in a sitting posture.

" *Fracture in the Middle of the Shaft.—(Vide Fig.* 4, *Plate IX.)*—When this portion of the bone is fractured, a shortening of the limb invariably takes place, which is produced by the action of those muscles which are attached to the whole length of the thigh-bone. The most usual position of the fractured extremities is, for the lower portion to be drawn upwards and inwards by the adductor muscles, while the upper is thrown outwards, forming in this situation a perceptible protuberance produced by the action of the m. gluteus maximus. This is not, however, the invariable direction; for if the fracture occurs midway between the insertion of the m. gluteus maximus and the external condyle, the m. vastus externus will draw both portions in such a direction as to form a salient angle outwards, in which case very little shortening occurs.

" *Transverse Fracture immediately above the Condyles.—(Vide Fig.* 5, *Plate IX.)*—In this case, the m. gastrocnemius externus plantaris and popliteus, draw downwards and backwards the inferior portion of the bone, so that the inferior extremity of the upper part appears to be the one displaced.

" *In Fractures of the Condyles*, the vasti muscles passing around them to be inserted into the patella, admit of but little displacement. They have a slight tendency to be drawn backwards by the m. gastrocnemius externus, and the inner one upwards by the tendon of the m. adductor magnus.

" *Fractures of the Thigh* may be considered of more difficult management than that of any other bone, both in consequence of the difficulty in keeping the parts in apposition, and of the violence necessary to produce solution of continuity.

" In all fractures, the causes of the displacement of the fractured portions of bone is muscle; and as coaptation is necessary for the cure, it becomes the object of the surgeon to place the limb in that situation best adapted mechanically to prevent the influence of muscles, as well as to subdue their irritability by constitutional means. For the first indication various apparatus have been invented, and numerous positions of the fractured limb recommended. It occurs to me, however, that no general practice, no constant rule can be laid down as the best. The surgeon's mind in every case can alone form the means to be adopted, as applicable to the individual instance; bearing in mind that coaptation must not only be produced, but preserved for a longer or shorter period. From the size of the femur, its reparation is not completed, under ordinary circumstances, in less than fifty days." 144.

